# Overexpression of the transcribed ultraconserved region Uc.138 accelerates colon cancer progression

**DOI:** 10.1038/s41598-021-88123-9

**Published:** 2021-04-21

**Authors:** Yuki Kuwano, Kensei Nishida, Kazuhito Rokutan

**Affiliations:** Department of Pathophysiology, Institute of Biomedical Sciences, Tokushima University Graduate School, 3-18-15 Kuramoto-cho, Tokushima, 770-8503 Japan

**Keywords:** Cancer, Cell biology, Molecular biology

## Abstract

Ultraconserved regions (UCRs) are 481 genomic sequences with 100% identity across humans, rats, and mice. Increasing evidence suggests that non-coding RNAs transcribed from UCRs are involved in various diseases, especially cancers. The human *transformer 2β* gene (*TRA2B*) encodes a UCR (uc.138) that spans exon 2 and its neighboring introns. *TRA2B4* RNA is the only transcript that contains the whole exon 2 among five spliced *TRA2B* RNA variants (*TRA2B1-5*). *TRA2B4* is upregulated in colon cancer cell lines, although it is not translated to Tra2β protein because of its nuclear retention. Nevertheless, the clinical significance and biological functions of uc.138 in colon cancer cells remain unclear. In this study, RNA in situ hybridization showed that *TRA2B4* was predominantly overexpressed in the nucleus of colon adenocarcinoma and adenoma. Overexpression of *TRA2B4* in colon cancer HCT116 cells promoted cell proliferation by changing the expression of G2/M-related cell cycle regulators. Moreover, *TRA2B4* increased migration and cell viability in a uc.138 sequence-dependent manner. *TRA2B4* significantly enhanced tumorigenesis in vivo. Taken together, uc.138 encoded in *TRA2B4* plays an oncogenic role in tumor progression and may become a potential biomarker and therapeutic target in colon cancer.

## Introduction

Ultraconserved regions (UCRs) are 481 segments that are absolutely conserved longer than 200-bp in length between orthologous regions in human, rat, and mouse genomes^1^. Transcripts that are transcribed from the genomic *loci* containing UCRs are categorized as a novel group of functional RNAs, transcribed-UCRs (T-UCRs)^2^. Recent genome-wide expression profiling studies have reported that T-UCRs are dysregulated in several types of human diseases such as chronic lymphatic leukemia, cervical, colorectal, lung, and breast cancers^3–8^.

Transformer 2β (Tra2β) contains two serine/arginine domains and an RNA recognition motif, and it functions as a sequence-specific pre-mRNA splicing enhancer^9, 10^. Overexpression of the Tra2β protein is associated with the development of several cancers^11–14^. We previously reported that the protein and mRNA levels of Tra2β were upregulated in colon cancer cells, and silencing of Tra2β decreased colon cancer cell proliferation^15^. Tra2β interacts with the 3′-UTR of the antiapoptotic *BCL2* mRNA and increases resistance to apoptosis^16^. Thus, Tra2β is supposed to act as an oncogenic protein to accelerate tumor progression^15,17^. The human *TRA2B* gene contains 10 exons and 9 introns and generates five mRNA isoforms (*TRA2B1* to *5*) through alternative splicing^18^. Among them, *TRA2B1* mRNA lacking exon 2, which contains multiple premature termination codons (PTCs), is translated into the Tra2β protein^18^. Intriguingly, the *TRA2B* gene encodes a 419-bp UCR (uc.138) with perfect human-to-rodent sequence identity^1^. The UCR in the *TRA2B* gene spans exon 2 and its neighboring introns. Among the five spliced transcripts, only *TRA2B4* contains a complete exon 2. In general, premature termination codon-containing RNAs such as *TRA2B4* are considered byproducts of aberrant splicing and are degraded by RNA surveillance mechanisms such as nonsense-mediated mRNA decay (NMD). However, we have previously reported that the RNA-binding protein Hu antigen R (HuR) increased *TRA2B4* production by upregulating the inclusion of exon 2 in response to oxidative stress^19^. In addition, *TRA2B4* was preferentially retained in the nucleus of colon cancer cells by association with a nuclear protein nucleolin, which helped to escape degradation through nonsense-mediated mRNA decay^20,21^. Using cDNA libraries prepared from 24 patients with colon cancer, we determined that colon cancer tissues expressed significantly higher levels of *TRA2B4* compared with surrounding normal tissues by qPCR^20^.

We also found that *TRA2B4* inhibited p21-mediated cellular senescence by interrupting the binding between Sp1 and the *CDKN1A* promoter in colon cancer cells^20^. Although several T-UCRs exhibit disease-specific expression and affect pathological conditions, the underlying biological functions of T-UCRs are largely unknown.

In the present study, we demonstrated RNA in situ hybridization and found that uc.138-containing *TRA2B4* was predominantly expressed in the nucleus of colon adenocarcinoma and adenoma cells. Then, we investigated the gene expression profiles in stable *TRA2B4*-overexpressed colon cancer cells and revealed that G2/M-related cell cycle regulators were significantly upregulated. Overexpression of the transcribed uc.138, as one of the spliced isoforms of *TRA2B*, promoted migration and cell proliferation of colon cancer cells. These results indicate that the ultraconserved uc.138 has oncogenic roles in the progression of colon cancer.

## Results

### Expression of *TRA2B4* in human colon adenocarcinoma

**Figure 1:**
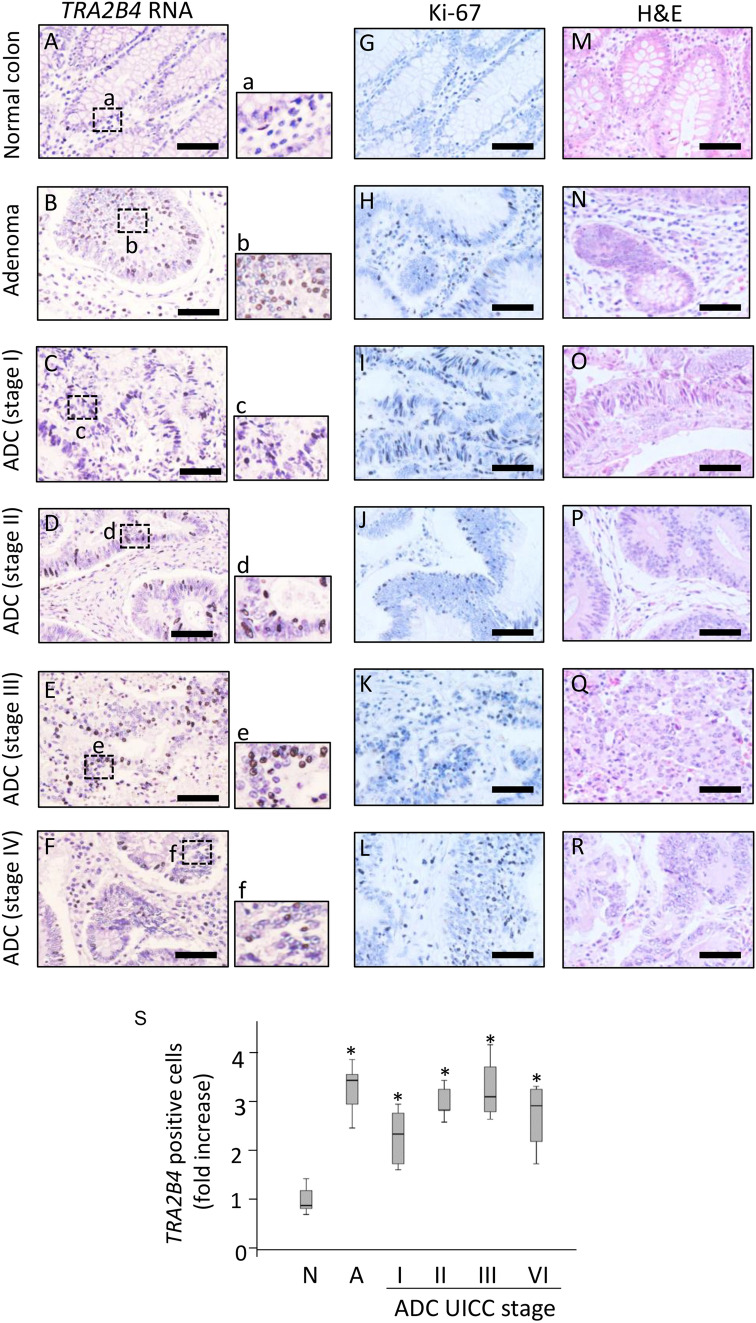
Expression of *TRA2B4* in normal colonic epithelium and colon tumor. (**A**–**F**) RNA in situ hybridization for the expression of *TRA2B4* was performed using normal colon (**A**), adenoma (**B**), and colon adenocarcinomas (ADC) (**C**–**F**) tissue microarray (TMA). Magnified views of these areas are shown with lowercase letters. DIG-labeled antisense probe targeting for exon 2 in *TRA2B4* was hybridized to normal colonic epithelium or primary tumor and then hematoxylin was used as the counterstain. (**G**–**L**) Immunohistochemical expression of Ki-67 in normal colon and colorectal cancer tissues. (**M**–**R**) Hematoxylin and eosin (H&E) stain in normal colon and colorectal cancer tissues. Scale bars: 50 μm. (**S**) A number of positive cells per area in each tumor stages (UICC stages I–IV), adenoma (A), and normal colon (N) were counted (n = 10 samples per group, with 10 fields per samples). *Significantly different by the unpaired Student’s *t* test compared with normal colon (*p* < 0.05).

The *TRA2B* gene (185,914,558–185,938,103 located on Chromosome 3) contains an UCR (uc.138: length 419 bp, 185,931,503–185,931,921) that spans exon 2 (ENSE00003574041: length 276 bp, 185,931,852–185,931,577) and neighboring introns^1,18^. Among the *TRA2B* mRNA isoforms, only *TRA2B4* includes exon 2 (Ensembl transcript ID: ENST00000456380.5.). First, we investigated whether the UCR-containing *TRA2B4* is expressed in human colon carcinoma tissues. *TRA2B4* expression was examined by RNA in situ hybridization targeting exon 2 using colon cancer tissue microarray (TMA) (Provitro, colon carcinoma tissues, no. 401 2211). TMA included 10 normal colon tissues, 10 adenomas, and 40 colon adenocarcinomas (International Union Against Cancer (UICC) stage I–IV). *TRA2B4* was weakly expressed in normal colonic epithelial cells (Fig. 1A). However, robust expression of *TRA2B4* was observed mainly in the nucleus of colorectal cancer cells (Fig. 1B–F). *TRA2B4* expression was detected at different clinical stages of colon adenocarcinoma tissues using in situ hybridization (Fig. 1C–F). Proliferative nuclear marker Ki-67 was stained to confirm proliferating colon cancer cells (Fig. 1G–L). *TRA2B4* was detected in Ki-67-positive colon cancer samples. Tissue arrays were stained with hematoxylin and eosin (H&E) to define the tumor region (Fig. 1M–R). A number of positive cells per area in each adenocarcinoma (ADC) tumor stages (UICC stages I–IV), adenoma (A), and normal colon (N) was measured. Although the levels of *TRA2B4* were not correlated with the severity of UICC stage, its expression was significantly higher in tumors than that in the normal colon tissue (Fig. 1S).

### Identification of uc.138-related gene expression profiles in colon cancer cells

**Figure 2:**
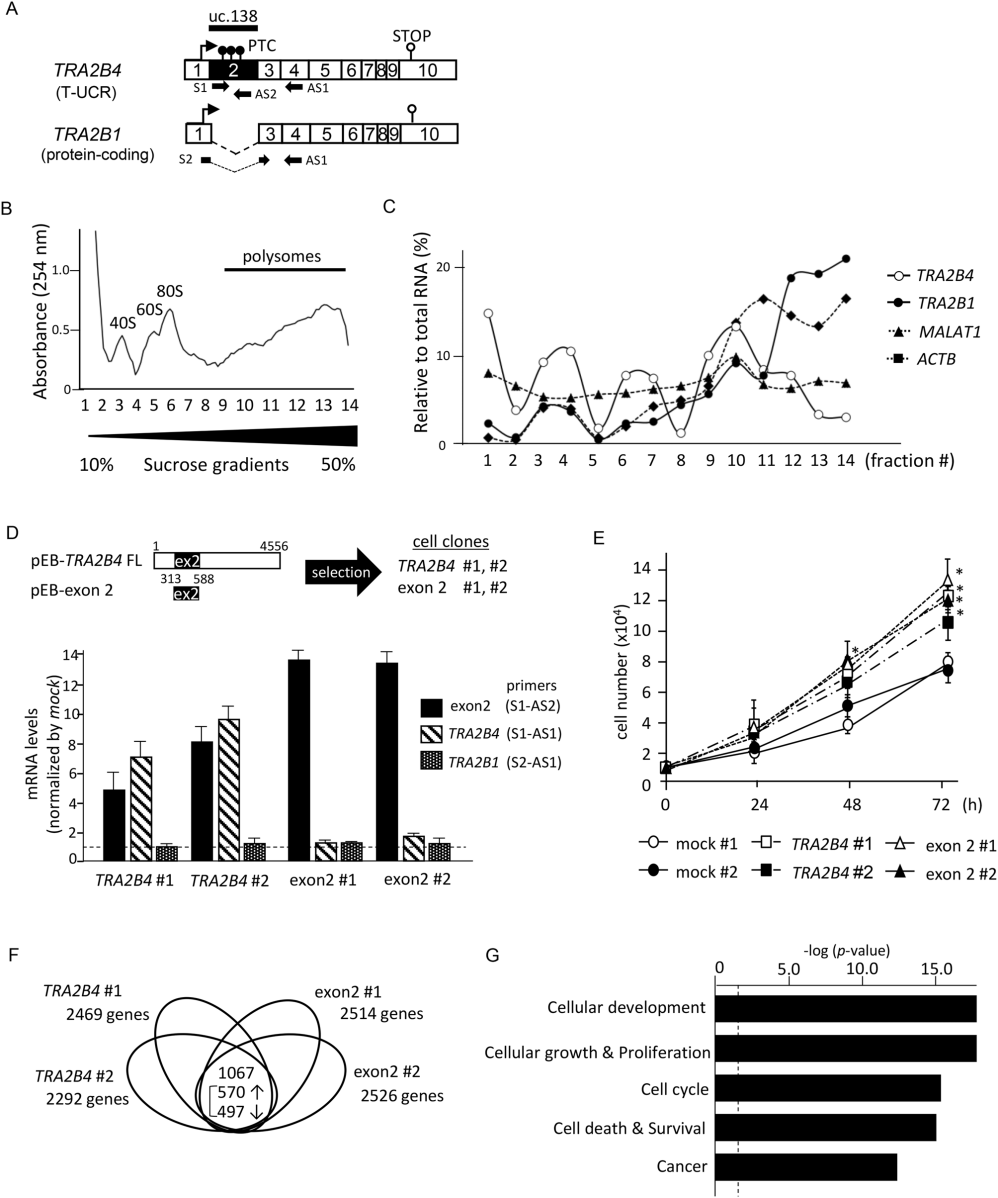
Identification of uc.138-related gene expression profiles in colon cancer cells. (**A**) Schematic diagram of the human *TRA2B* gene. Exons are indicated by open boxes and Arabic numbers. Filled boxes denote the ultraconserved uc.138. Two major splice variants *TRA2B1* and *TRA2B4* are generated from the *TRA2B* gene and the use of each exon is shown. Black arrows show the specific primers used to detect each of the transcripts. Arrows in exon 1 indicate a translation start site. PTCs; premature termination codons. (**B**) The absorbance at 254 nm was measured during the fractionation of polysomes. (**C**) The abundance of *TRA2B4, TRA2B1*, *ACTB*, and *MALAT1* in each fraction from the polysome profiling was determined by qPCR. (**D**) Relative expression levels of *TRA2B1*, *TRA2B4*, and exon 2 in human colorectal carcinoma (HCT116) cells that were stably transfected with pEB-multi vector encoding *TRA2B4* (exon 1–exon 10) or exon 2 were measured by RT-qPCR. *GAPDH* was used as an endogenous control. Data are expressed as the mean fold changes ± standard deviation (SD; n = 6), compared with those in the mock-treated cells. (**E**) Effects of stable overexpression of *TRA2B4* or exon 2 on cell proliferation were monitored by counting the number of cells at the indicated times. Values are mean ± SD from five independent experiments. *Significantly different by the unpaired Student’s *t* test compared with mock #1-treated cells (*p* < 0.05). (**F**) The number of differentially expressed genes in *TRA2B4* or exon 2-overexpressed cells (≥ 1.5-fold), compared with mock #1-treated cells. (**G**) Commonly regulated 1067 genes were subjected to Ingenuity Pathway Analysis (QIAGEN Bioinformatics) to identify biologically relevant functions.

Uc.138-containing *TRA2B4* transcript encodes multiple PTCs in exon 2 (Fig. 2A). RNA variants with PTCs are preferentially subjected to NMD as aberrant RNAs and are not translated into proteins. To test whether *TRA2B4* has protein-coding potential, we performed polysome profiling using sucrose gradient-based fractionation. Glucose gradient centrifugation fractionated the nonpolysome-associated (fractions 1–2), small (40S) and large (60S) ribosomal subunits, monosomes (80S) (fractions 3–8), and progressively larger polysomes (fractions 9–14) (Fig. 2B). The relative distribution of *TRA2B4*, *TRA2B1* (Tra2β protein-coding transcript), *ACTB*, and non-coding RNA *MALAT1* on polysome gradients was measured by qPCR. *TRA2B1* and *ACTB* were enriched in the high molecular weight polysome fractions, whereas *TRA2B4* and *MALAT1* were not abundant in the polysome fractions, indicating that *TRA2B4* did not interact with the translational machinery (Fig. 2C). To investigate the molecular mechanism underlying the influence of uc.138 expression, we first established stable cell lines that overexpressed full-length *TRA2B4* (ex 1–ex 10) or *TRA2B* exon 2 encoding uc.138 (Fig. 2A). We introduced pEB-Multi constructs to human colorectal carcinoma (HCT116) cells and selected cells that stably expressed high levels of *TRA2B4* or *exon2* (*TRA2B4* #1, *TRA2B4* #2, exon2 #1, and exon2 #2) (Fig. 2D). The expression levels of *TRA2B4* (primer S1-AS1), exon 2 (S1-AS2), and *TRA2B1* (S2-AS1) were confirmed using real-time quantitative (q) PCR with specific primers, as shown in Fig. 2A. The expression levels of *TRA2B4* and exon 2 were significantly increased in the stable lines, whereas the expression of another spliced isoform, *TRA2B1*, was not affected (Fig. 2D). We previously showed that expression levels of *TRA2B1* did not affect *TRA2B4* expression in HCT116 cells^21^. The introduction of *TRA2B4* and exon 2 significantly increased cell growth (Fig. 2E). As shown in Fig. 2F, microarray-based global expression analysis showed that *TRA2B4* #1 and *TRA2B4 #2* stable cells differentially expressed 2469 and 2292 genes in total, compared with those in the mock-treated control cells, respectively (≥ 1.5-fold) (Fig. 2D) (NCBI Gene Expression Omnibus #GSE161169). We found that expression levels of 1354 genes, including 693 upregulated genes and 681 downregulated genes, were commonly altered in both *TRA2B4* #1 and #2 overexpressing cells, compared with mock cells (≥ 1.5-fold). The cells specifically overexpressing exon 2 #1 and exon 2 #2, commonly altered the expression of 1880 genes, including 1178 upregulated genes and 702 downregulated genes (≥ 1.5-fold). Finally, we identified 1067 commonly altered genes among genetically diverse cells, *TRA2B4* #1, *TRA2B4* #2, exon2 #1 and exon2 #2, which were compared with mock cells (≥ 1.5-fold) (Fig. 2F, Supplementary Table 2). The differentially expressed genes were analyzed using ingenuity pathway analysis (IPA, Qiagen Bioinformatics) software to identify biological functions relevant to these genes. The enriched biofunctions of the genes regulated in uc.138-overexpressing cells were (1) cellular development (*p* = 9.05E−18), (2) cell growth and proliferation (*p* = 9.05E−18), (3) cell cycle (*p* = 6.55E−15), (4) cell death and survival (*p* = 8.19E−15), and (5) cancer (*p* = 2.04E−12) (Fig. 2G). These results suggest that uc.138 in exon 2 may facilitate proliferation of colon cancer cells.

### Uc.138 increases cell cycle progression in colon cancer cells

**Figure 3:**
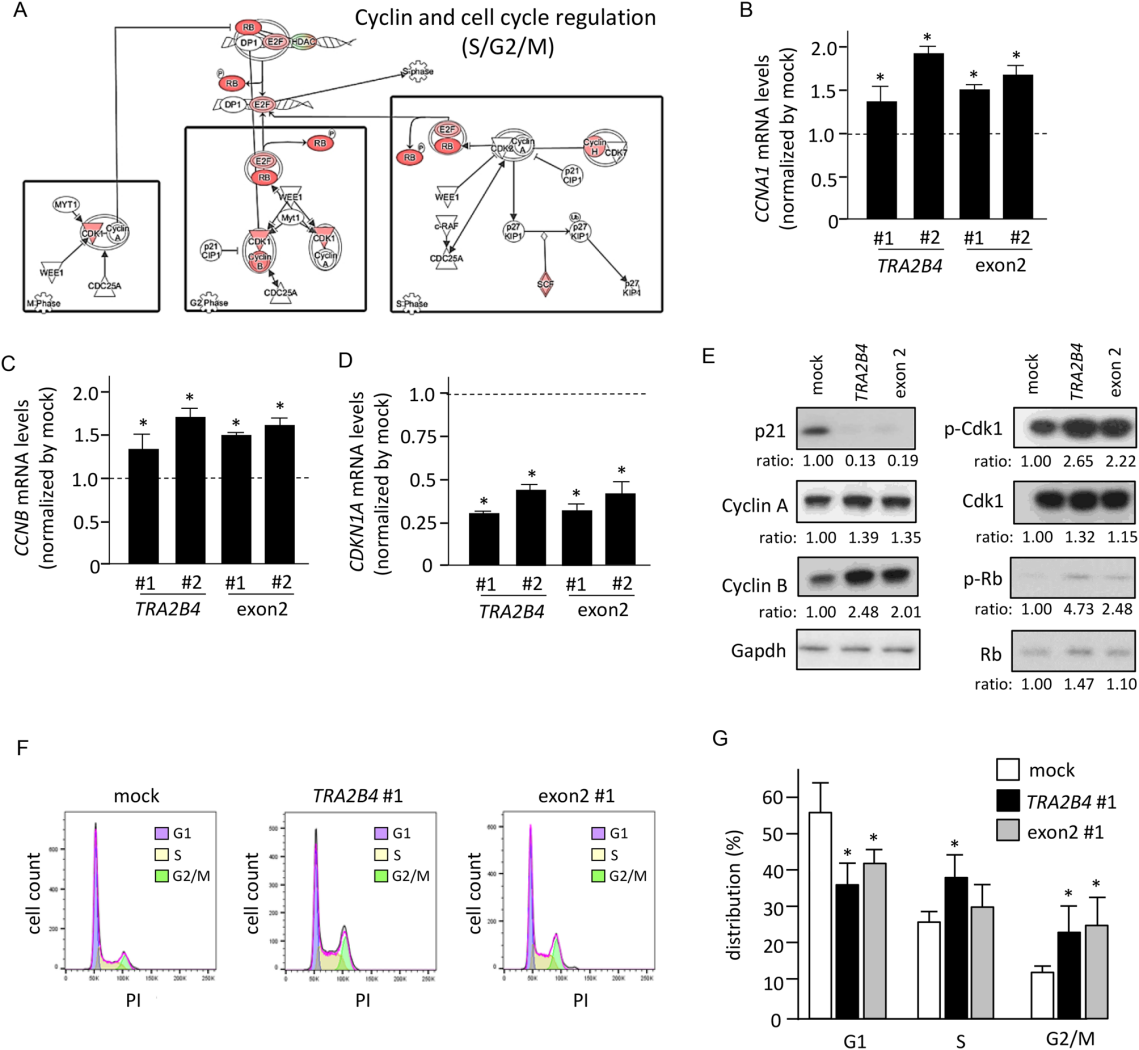
Effects of uc.138 on cell cycle progression in colon cancer cells. (**A**) Canonical pathways for cell cycle regulation represented by the differentially expressed genes between uc.138 overexpression and mock-transfected cells were analyzed using Ingenuity Pathway Analysis (QIAGEN Bioinformatics). The genes up- and down-regulated in uc.138 overexpressed cells are shown in red and green, respectively. (**B**–**D**) RNA levels of *CCNA1*, *CCNB*, and *CDKN1A*, respectively, were determined using RT-qPCR in *TRA2B4* or exon 2-overexpressed cells using *GAPDH* mRNA as an endogenous quantity control. Values are mean ± SD (n = 6). *Significantly different by the unpaired Student’s *t* test (*p* < 0.05). (**E**) Amounts of cell cycle related proteins and phosphorylation of Cdk1 and Rb were determined using western blotting with Gapdh as a loading control. (**F**) Effects of *TRA2B4* or exon 2-overexpression on cell cycle distribution were measured by flow cytometry. (**G**) The quantitative statistics of cell number in each phase. Values are means ± SD (n = 6). *Significantly different by the unpaired Student’s *t* test (*p* < 0.05).

We analyzed whether dysregulation of the cell cycle caused abnormal proliferation of uc.138-overexpressing cells. The canonical pathway of ‘Cyclin and Cell cycle regulation’ was represented by differentially expressed 1067 genes identified by microarray analysis (Fig. 3A). The molecules encoded by up- or down-regulated genes are shown in red and green, respectively, in S/G2/M phases (Fig. 3A). Overexpression of uc.138 increased G2 and S phases-related gene expression, including CDK1, cyclin A, and cyclin B. Real-time PCR analysis showed that *CCNA1* and *CCNB2* mRNA levels were significantly increased in both *TRA2B4* and exon 2-introducing cells (Fig. 3B,C). In contrast, the expression levels of *CDKN1A* encoding p21, an inhibitor of cyclin-dependent kinases, were reduced (Fig. 3D). Protein levels of cyclin A and cyclin B were increased, whereas p21 expression was down-regulated in these cells (Fig. 3E). We next confirmed that phosphorylation of mitotic protein kinase CDK1 was increased in these cells (Fig. 3E). The phosphorylation of cell cycle regulator Rb was elevated in *TRA2B4* and exon 2-overexpressing cells. To investigate the possibility that uc.138 contributes to elevated cell proliferation, we investigated whether *TRA2B4* or exon 2 expression in colon cancers induced cell cycle progression (Fig. 3F). Using cytofluorimetry, we found that overexpression of *TRA2B4* or exon 2 was associated with an increase in the percentage of G2/M cells (Fig. 3G). These results suggested that overexpression of uc.138 significantly increased cell proliferation via upregulation of cell cycle-regulatory proteins.

### Uc.138 overexpression affects resistance to apoptosis

**Figure 4:**
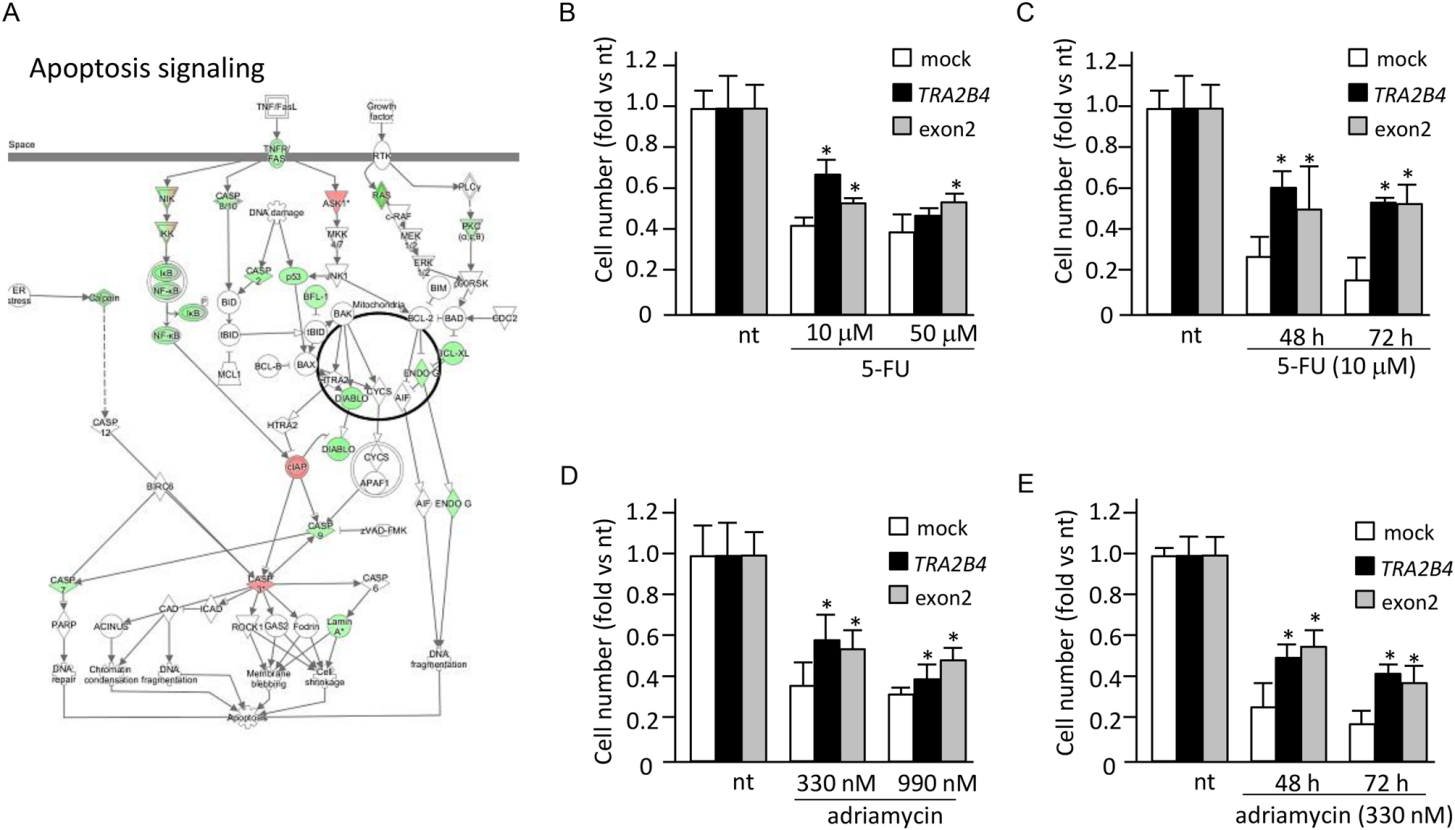
Effect of uc.138 on susceptibility to an anticancer drug. (**A**) Canonical pathways for “apoptosis signaling” represented by the differentially expressed genes between uc.138 overexpression and mock-transfected cells were analyzed using Ingenuity Pathway Analysis (QIAGEN Bioinformatics). The genes up- and down-regulated in uc.138 overexpressed cells are shown in red and green, respectively. (**B**,**D**) Human colorectal carcinoma (HCT116) cells with stable overexpression of *TRA2B4* or exon 2 were exposed to 5-fluorouracil (5-FU) or adriamycin for 24 h at indicated concentrations. Subsequently, growing cells were harvested and counted. (**C**,**E**) The number of cells with stable overexpression of *TRA2B4* or exon 2 was measured after exposure to 10 μM 5-FU or 330 nM adriamycin at different time-points (0, 48 and 72 h). nt: non-treatment. Values are mean ± SD from four independent experiments. *Significantly different by the unpaired Student’s *t* test compared with mock-treated cells (*p* < 0.05).

According to gene expression data from microarray analysis, one of the biofunctions enriched by differentially expressed genes was ‘cell death and survival’ (*p* = 8.19E−15) (Fig. 2A). Ingenuity pathway analysis showed that cell death-related genes were significantly down-regulated in uc.138-introduced cells (Fig. 4A). To further verify the cellular function of uc.138, we investigated whether the expression levels of uc.138 influenced the susceptibility to apoptotic cell death. As shown in Fig. 4B,D, overexpression of *TRA2B4* and exon 2 significantly inhibited 5-fluorouracil (5-FU)- or adriamycin-induced cell death after 24 h treatment. Compared with mock-treated cells, the cell viability of *TRA2B4* or exon 2-introduced cells were significantly increased at different time points (Fig. 4C,E). These results indicated that expression of uc.138 affected tumorigenic properties such as resistance to anticancer drugs in colon cancer cells.

### Mutation of uc.138 impaired cell proliferation

**Figure 5:**
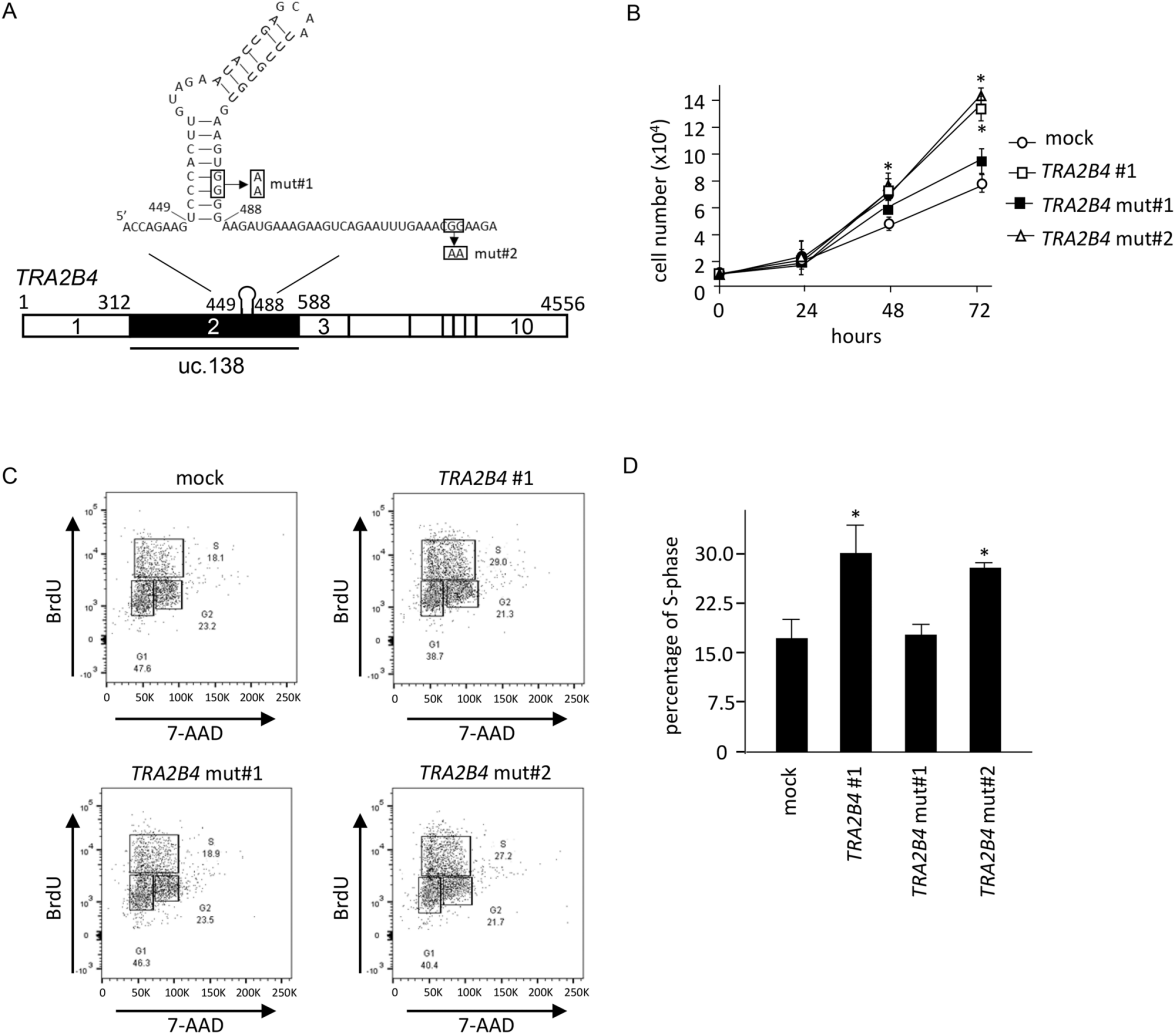
Effect of mutations in uc.138 on cell proliferation. (**A**) Nucleotide sequence of uc.138-encoding *TRA2B4* exon 2. Predicted stem-loop structure (449–488 nt), the mutations of 485-GG-486 to 4865-AA-486 (mut#1) and 515-GG-516 to 515-AA-516 (mut#2) are indicated in the schema. (**B**) Effects of stable overexpression of *TRA2B4* or *TRA2B4* mutations on growth of human colorectal carcinoma (HCT116) cells were monitored by counting the number of cells at the indicated times. Values are mean ± SD from five independent experiments. *Significantly different by the unpaired Student’s *t* test compared with mock #1-treated cells (*p* < 0.05). (**C**) BrdU incorporation was analyzed using flow cytometry. Scatter plots of fluorescence intensities of BrdU incorporation (y-axis) against DNA content (x-axis) are shown. The numbers depict percentages of cells in S phase. 7-AAD; 7-amino-actinomycin D. (**D**) The quantitative statistics of percentage of the S phase. Values are mean ± SD (n = 3). *Significantly different by the unpaired Student’s *t* test compared with mock (*p* < 0.05).

In a previous study, we identified that uc.138 contains a stem-loop structure (449–488 nt) using the programs CentroidFold (http://rtools.cbrc.jp/centroidfold/) and M-FOLD (http://www.unafold.org/)^20^. Transient expression of *TRA2B4* significantly decreased p21 levels and accelerated cell growth; however, the introduction of mutations in the stem-loop motif canceled these effects^20^. In this study, we established stable cells that overexpressed *TRA2B4* with/without mutations and investigated the effect of uc.138 on the cell cycle. The Mfold program predicts a ΔG value of − 13.80 kcal/mol for wild-type stem-loop structure. As shown in Fig. 5A, two-point mutations were introduced to disrupt stem-loop formation (mut#1: 485-GGGG-488 to 485-AAGG-488, ΔG value of − 5.90 kcal/mol). Cells overexpressing *TRA2B4* with mutations that did not influence the stem-loop structure (mut#2: 564-GGGG-567 to 564-AAGG-567, ΔG value of − 12.90 kcal/mol) were also generated. Stable overexpression of *TRA2B4* or *TRA2B4* mut#2 accelerated cell proliferation, whereas *TRA2B4* mut#1 did not affect the proliferation rate (Fig. 5B). To measure the population of cells in the S phase, BrdU incorporation in vivo was analyzed by flow cytometry (Fig. 5C). The percentage of BrdU-positive cells was significantly increased by stable overexpression of *TRA2B4* or *TRA2B4* mut#2 (Fig. 5D); however, the expression of *TRA2B4* mut#1 did not change BrdU incorporation. These results suggested that uc.138 was involved in the progression of the cell cycle in a sequence-dependent manner. We analyzed the subcellular localization of *TRA2B4* wild-type and mutations. Corresponding with the cellular function, *TRA2B4* wild-type and mut#2 transcripts were predominantly located in the nuclear of HCT116 cells (Supplementary Fig. S1). In contrast, mut#1 was more abundant in the cytoplasmic fraction (Supplementary Fig. S1). Using biotinylated RNA pull-down assays followed by LS/MS analysis, we reported that *TRA2B4* interacted with several nuclear proteins such as Sp1, nucleolin, hnRNPA, and hnRNPU via exon 2^21^. Thus, decreased nuclear expression of mut#1 may cause loss of cellular functions thorough defects of association with nuclear factors.

### Uc.138 promotes colon cancer cell migration and tumor growth

**Figure 6:**
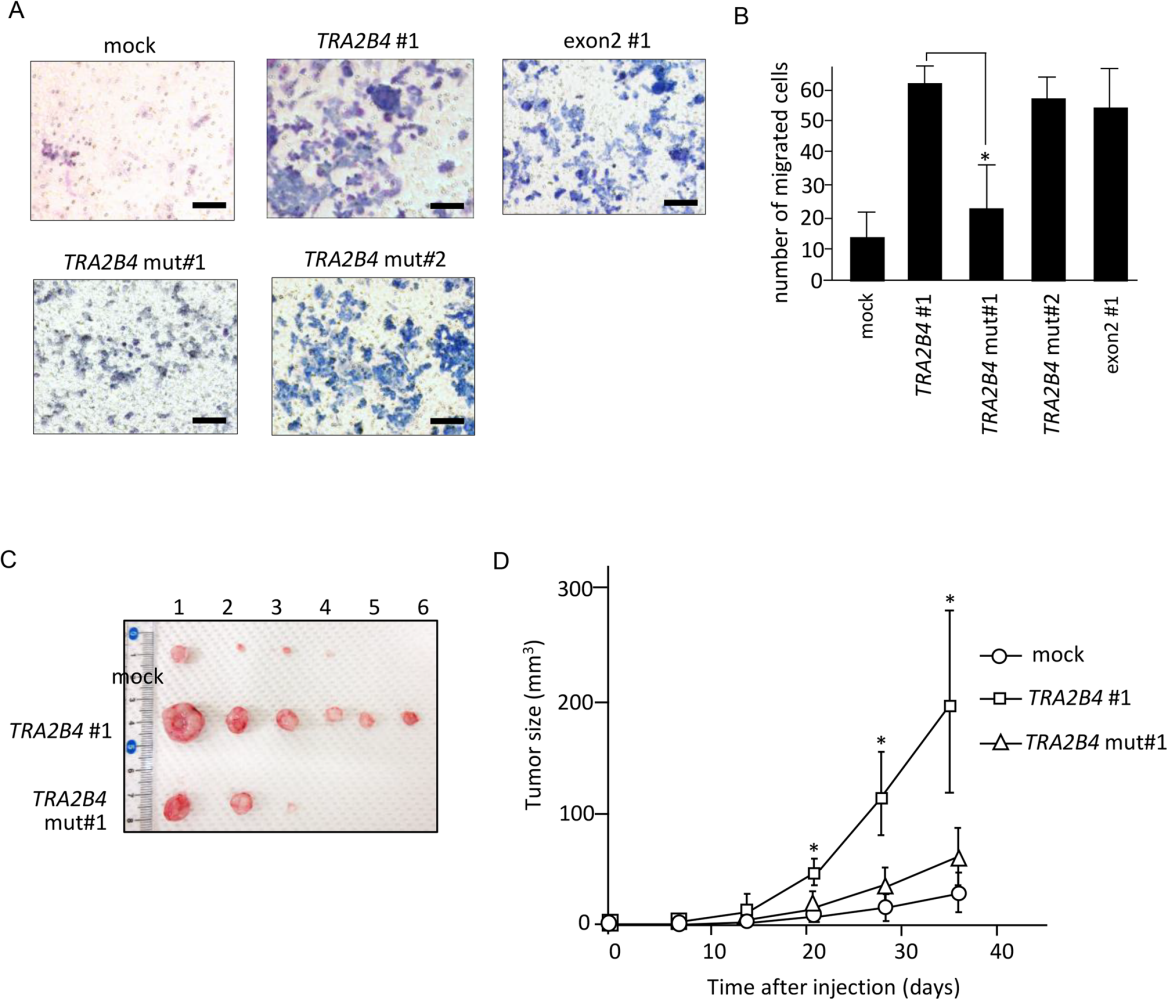
Effect of uc.138 on cell migration and tumor progression. (**A**) Transwell migration assays were performed using human colorectal carcinoma (HCT116) cells with stable overexpressed *TRA2B4*, exon 2, *TRA2B4* mut#1, or *TRA2B4* mut#2. Migrated cells were stained by a Diff-Quick staining kit. (**B**) Quantification of cell migration was expressed by cell counting. Values represent mean ± SD (n = 8). *Significant difference versus *TRA2B4* #1 (unpaired Student’s *t* test, *p* < 0.05). (**C**) Images of the tumors from the mice injected with cells which were stably overexpressed *TRA2B4*, *TRA2B4* mut#1, or mock. (**D**) Tumor size in nude mice injected with HCT116 cells was measured every 7 days. Values represent mean ± SD (n = 6). *Significant difference versus mock (unpaired Student’s *t* test, *p* < 0.05).

IPA of the 1067 differentially expressed genes showed Cellular Movement (P = 1.93E−03–4.86E−09) and Cell Morphology (P = 1.70E−03–6.66E−09) as significantly affected molecular and cellular functions. We tested cell migration as one of uc.138-related malignant phenotypes of cancer cells. To investigate whether uc.138 influences cancer cell migration, we performed transwell assays. Elevated expression of *TRA2B4*, exon 2, and *TRA2B4* mut#2 caused an increase in cell migration (Fig. 6A,B). The overexpression of *TRA2B4* mut#1 did not change migration activity.

To confirm the effect of uc.138 on tumor progression, we constructed a xenograft tumor model by injection of mock, *TRA2B4-* or *TRA2B4* mut#1-overexpressed cells. When compared with mock injection, the *TRA2B4* overexpressed cells exhibited a rapid rate of tumor growth as measured by tumor volumes (Fig. 6C,D). In contrast, injection of *TRA2B4* mut#1-overexpressed cells did not accelerate tumor progression. We found that overexpression of *TRA2B4* wild-type and mut#2 suppressed apoptosis induced after treatment of anticancer agents. Introduction of mut#2 did not affect resistance to apoptosis (Supplementary Fig. S2). Taken together, these results suggest that uc.138 enhances cell migration and tumor growth in colon cancer.

## Discussion

There are 481 completely conserved sequences (referred to as UCRs) between the ortholog regions of human, mouse, and rat genomes^1^. Transcripts generated from UCRs (T-UCRs) are defined as a novel category of functional RNAs. Increasing evidence has indicated that aberrant expression of T-UCRs is shown in various types of cancers, and several T-UCRs act as oncogenes or tumor suppressors^6^. In this study, we show that uc.138, which is encoded in the *TRA2B* gene, is upregulated in human colon cancer tissues using RNA in situ hybridization. We assessed the effects of increased uc.138 expression on biological pathways by measuring changes in gene expression in stable uc.138-overexpressing cells. Stable overexpression of uc.138 in colon cancer cells significantly increased cell proliferation. Pathway analysis suggested that cancer-related pathways, including cellular development, cell proliferation, cell cycle, and cell death, were influenced by differentially expressed genes in uc.138-overexpressed cells. Stable overexpression of uc.138 increased cell migration in vitro and tumor formation in an in vivo murine xenograft model. Interestingly, the host gene of uc.138, *TRA2B*, transcribes five spliced variants (*TRA2B1*-*TRA2B5*)^9^. Among them, *TRA2B4*, which includes uc.138-containing exon 2, is retained in the nucleus and is not translated into the Tra2β protein^21^. *TRA2B1* mRNA, which skips exon 2, can translocate to the cytoplasm and generate functional Tra2β protein. We previously reported that *TRA2B1* mRNA and Tra2β were upregulated in colon cancer tissues^15^. Overexpression of Tra2β reduced colon cancer cell death by stabilizing the *anti-apoptotic factor BCL2*^16^. These results suggest that different RNA variants, such as *TRA2B1* and the functional RNA *TRA2B4,* can regulate cellular function through multiple pathways. Therefore, dysregulation of the *TRA2B* gene may be associated with promotion of tumorigenesis by not only Tra2β protein but also T-UCR uc.138.

Increasing evidence indicates that T-UCRs control physiological status and homeostasis, such as epithelial differentiation and renewal of the intestinal mucosa. For instance, uc.291 interacts with the chromatin remodeling protein ACTL6A and promotes epithelial differentiation^22^. Uc.173 is directly associated with and destabilizes pri-miR-195, resulting in the stimulation of intestinal epithelial renewal^23^. Uc.173 has an important role in intestinal epithelial barrier function by inhibiting the miR-29b/tight junction protein claudin-1 axis^24^. According to the recent studies about functions mediated by RNA-RNA interactions, we predicted microRNAs which were specifically associated with uc.138. We found that two putative miRNA target sites (miR-3925 and miR-6881, the minimum free energy of binding was − 16.4 and − 18.3 kcal/mol, respectively) in uc.138 using miRBase (http://www.mirbase.org/) and RNAhybrid (https://bibiserv.cebitec.uni-bielefeld.de/rnahybrid). Although the functions of these miRNAs have not been reported yet, uc.138 sequences may contain the potential to post-transcriptionally interact with miRNAs.

Thus, dysregulation of T-UCRs in the intestinal epithelium may be associated with the development of diseases. In fact, the expression levels of uc.261 were elevated in intestinal mucosa in patients with Crohn’s disease and positively correlated with disease activity index and histological index^7^. Zhang et al. reported that uc.338 was upregulated in human colorectal cancer and increased cell proliferation by activation of the PI3K/AKT pathway^25^. The methylation status and expression levels of uc.160, uc.283, and uc.346 were dysregulated in neoplastic tissues from colorectal cancer, and uc.160 and uc.346 enhanced colorectal cancer cell progression^26^.

Although aberrant expression of T-UCRs occurs in many types of diseases, how T-UCRs contribute to cellular functions in general is still unclear. To date, several studies have shown that T-UCRs can regulate gene expression by binding to target proteins or mRNAs in a sequence-dependent manner. We previously reported that silencing of *TRA2B4* inhibited cell growth through induction of p21/*CDKN1A* by accelerating interaction between Sp1 and the *CDKN1A* promoter^20^. Human fibroblast cell line (TIG-3) exhibited an age-associated reduction of *TRA2B4* and expression levels of *TRA2B4* and *CDKN1A* were negatively correlated^20^. Here, cell proliferation assay using stable cell lines suggested that p21 was one of the key regulators of uc.138-repated cell cycle progression. In non-small lung cancer cells, uc.339 functioned as a decay for microRNAs that targeted Cyclin E2 and promoted cell growth and migration^27^. Overexpression of uc.338 in cervical cancer cells promoted lymph node metastasis by binding to the 3′-UTR of tissue inhibitor of metalloproteinase 1^28^. Uc.323 is associated with a histone methyltransferase EZH2 and decreased transcription of cardiac hypertrophy-related CPT1b, which ameliorated cardiac hypertrophy^29^.

In this study, we demonstrated that uc.138 was significantly upregulated in colon cancer tissues. Overexpression of uc.138 was associated with an increase in cell proliferation, migration, and tumor progression. Further studies are needed to reveal the pathological signatures that influence the aberrant expression of *uc.138*. Our previous study showed that the transcription of the *TRA2B* gene encoding uc.138 is upregulated by activation of HSF1 in response to oxidative stress in human colon cancer cells^15^. Since *TRA2B4* contains PTCs, *TRA2B4* is supposed to be degraded by NMD as an aberrant RNA. The expression levels of *TRA2B4* were significantly lower than that of *TRA2B1* in normal colon epithelial cells^20^. Oxidative stress-induced phosphorylation of HuR leads to aberrant alternative splicing of *TRA2B* pre-mRNAs and increases *TRA2B4* generation in colon cancer cells^19^. In addition, *TRA2B4* was protected in the nuclear by association with Nucleolin in colon cancer^21^. These posttranscriptional regulation of *TRA2B4* might be the reason why *TRA2B4* showed more significant increase in cancer cells as compared with *TRA2B1.* Altered expression of T-UCRs caused by cellular stimuli can disrupt homeostasis within tissue microenvironments. Taken together, these results suggest that tumorigenic T-UCR uc.138 may become a potential biomarker and novel therapeutic target in patients with colon cancer.

## Materials and methods

### Cell culture and transfection

Human colon cancer cell lines HCT116 (CCL-247) were obtained from American Type Culture Collection (ATCC) (Manassas, VA) and cultured in McCoy's 5A medium (Thermo Fisher Scientific, Waltham, MA) supplemented with 5% (v/v) heat-inactivated fetal bovine serum and antibiotics (penicillin and streptomycin) at 37 °C in 5% CO_2_. The human full-length *TRA2B4* (exon 1 to exon 10) and exon 2 were amplified using PCR with the primer set listed in Supplementary Table 1. The amplified products were separated with a gel extraction kit (Qiagen, Hilden, Germany) and cloned into the mammalian expression vector pEB-Multi-Bsd (Wako, Osaka, Japan) using XhoI and EcoRV sites. Point mutations were introduced into pEB-Multi *TRA2B4* vector using a site-directed mutagenesis kit (KOD-Plus-Mutagenesis Kit; Toyobo, Osaka, Japan). The construct sequence was confirmed by DNA sequencing. HCT116 cells were then transfected with either empty, *TRA2B4*, or an exon 2-expression vector using Lipofectamine 2000 (Thermo Fisher Scientific) according to the manufacturer’s protocol. The stably transfected cells were selected using medium containing 5 µg/mL of Blasticidin S. (InvivoGen, San Diego, CA) for ~ 7 days.

### Western blotting

Whole cell lysates were prepared using RIPA buffer (Thermo Fisher Scientific) with a complete protease inhibitor cocktail (Roche, Mannheim, Germany) as previously described^15^. Ten micrograms of the extracted proteins were separated using sodium dodecyl sulfate-polyacrylamide gel electrophoresis and then transferred to a polyvinylidene fluoride membrane (Bio-Rad, Hercules, CA, USA). After blocking with 5% nonfat dry milk (Cell Signaling Technology, Danvers, MA, USA) for 1 h at room temperature, the membrane was incubated with anti-p21 (Santa Cruz Biotechnology, Santa Cruz, CA); anti-cyclin A, anti-cyclin B, anti-Rb, anti-phospho-Rb, anti-phospho-Cdk1, and anti-Cdk1 (Cell Signaling Technology); or anti-glyceraldehyde 3-phosphate dehydrogenase (Gapdh) (Santa Cruz) antibody overnight at 4 °C. Following incubation with an appropriate secondary antibody for 1 h at room temperature, the bound antibodies were detected with Pierce Western Blotting Substrate (Thermo Fisher Scientific). The intensities of the bound antibodies were quantified using ImageJ (National Institutes of Health, USA).

### qPCR

Total RNA was extracted from cells using RNAiso Plus (Takara, Tokyo, Japan) according to the manufacturer’s protocol. Isolated RNAs were reverse transcribed using ReverTra Ace qPCR RT Master Mix (Toyobo). The expression levels of *TRA2B1*, *TRA2B4*, *CCNA1*, *CCNB2*, *CDKN1A*, *ACTB*, and *GAPDH* mRNA were measured using specific primer sets and SYBR Green Master Mix (Thermo Fisher Scientific) (Supplementary Table S1).

### RNA in situ hybridization

The colon cancer TMA was provided by Provitro (Berlin, Germany; colon carcinoma tissues, no. 401 2211). Digoxigenin-labeled antisense RNA complementary to exon 2 (313–588 nt) of *TRA2B4* was generated using a DIG RNA labeling kit (Roche). The TMA sections were deparaffinized in xylene and then hydrated in graded concentrations of ethanol for 5 min each. These sections were incubated with 3% hydrogen peroxide and then hybridized at 55 °C with the RNA probes at 200 ng/mL overnight. After hybridization, tissue sections were washed in 5 × saline-sodium citrate (SSC), 50% formamide, and again with 0.5 × saline-sodium citrate. Then, the sections were incubated with 1:500 of an anti-digoxigenin antibody (Roche) overnight at 4 °C. After washing with TBS-T, sections were incubated with avidin–biotin complex solution (horseradish peroxidase-streptavidin–biotin complex, Vectastain ABC kit; Vector Laboratories) according to the manufacturer’s protocol. The final signal was developed with diaminobenzidine solution, and the tissues were counterstained with hematoxylin for 15 s. The signals were classified as negative (−) or positive (+) and counted.

### Polysome profiling

For the isolation of polysome fractions and polysome profiling, 10 × 106 HCT116 cells were pretreated with cycloheximide at a final concentration of 100 µg/mL for 10 min. Cytoplasmic lysates were prepared using lysis buffer (20 mM Tris–HCl, pH 7.5, 100 mM KCl, 5 mM MgCl2, 0.3% IGEPAL, and protease inhibitors). Cytoplasmic extracts were fractionated by ultracentrifugation through 10–50% linear sucrose gradients. For measurements of RNA distributions, 14 fractions were collected, and RNA was extracted with RNAiso Plus (TaKaRa).

### Cell migration assay

Cell migration was examined using transwell chambers (Becton-Dickinson Biosciences, Franklin Lakes, NJ, USA) as previously described^30^. In brief, after serum starvation for 48 h, cells were seeded in serum-free media on the upper side of a transwell chamber. The cells were allowed to migrate towards media containing 10% fetal bovine serum in the lower chamber. After incubation for 24 h, migrating cells were fixed and then stained with Diff-Quick stain (Sysmex, Kobe, Japan). The numbers of migrated cells were estimated as the average number of 5 random fields at 20 × magnification.

### Cell cycle analysis

The cells were harvested and fixed in 70% ethanol. After washing with phosphate-buffered solution, the cells were treated with 5 μg/mL of RNAse A and then stained in a solution containing 10 μg/mL of propidium iodide (Sigma-Aldrich). After 30 min staining, the samples were analyzed by flow cytometry using a BD FACSVerse (BD Biosciences). BrdU incorporation in vivo was measured by flow cytometry using the APC BrdU Flow Kit (BD Biosciences) in accordance with the manufacturer’s instructions.

### Microarray analysis

Total RNA was extracted from cells using a RNeasy kit (Qiagen) according to the manufacturer’s protocol. Purified RNA quality was assessed using an Agilent 2100 Bioanalyzer with an RNA 6000 Nano Labchip kit (Agilent Technologies, Santa Clara, CA, USA). RNA samples with > 9.0 RNA integrity number were used for further experiments. Expression profiles were measured using a whole human genome microarray (SurePrint G3 Human; Agilent). The expression data were analyzed using GeneSpring 14.9 (Agilent). Differentially expressed genes were subjected to Ingenuity Pathway Analysis (Qaigen Bioinformatics) to identify biologically relevant functions.

### Mouse xenograft experiments

Seven-week-old male athymic nude mice (Nippon SLC, Japan) were acclimated for 1 week. Mock, *TRA2B4*, or *TRA2B4#1*-overexpressed HCT116 cells (2 × 10^6^) were suspended in 100 μL phosphate-buffered solution and injected subcutaneously into the flanks of nude mice. Tumor volumes were examined every 7 days after transplantation. Four weeks after the injection, the mice were sacrificed, and the tumor volumes (length × width^2^ × 0.5) were measured.

All animal procedures conformed to the National Institutes of Health Guidelines for the Care and Use of Laboratory Animals. The experiments were carried out in accordance with the Guidelines for Animal Experiments of Tokushima University approved by Institutional Animal Care and Use Committee of Tokushima University (Approval number: T2820). All animal experiments were performed in accordance with the Animal Research: Reporting of In Vivo Experiments (ARRIVE) guidelines (https://arriveguidelines.org/arrive-guidelines).

### Statistical analysis

All statistical analyses were performed using SPSS 21.0 (SPSS Inc., Chicago, IL, USA). Results are expressed as mean ± SD. Significant differences between two groups were estimated by two-tailed Student’s *t* test. Non-parametric data were analyzed using the Wilcoxon–Mann–Whitney *U* test when comparing two groups. *p < 0.05 was considered statistically significant.

## Supplementary Information

Supplementary Information 1.

Supplementary Information 2.

## Abbreviations

*TRA2B*: Transformer 2-beta
UCR: Ultraconserved region
T-UCR: Transcribed-ultraconserved region
qPCR: Real-time quantitative polymerase chain reaction
