# Supplementary material for: Overexpression of the transcribed ultraconserved region Uc.138 accelerates colon cancer progression
Source: Sci Rep. 2021 Apr 21;11:8667. doi: 10.1038/s41598-021-88123-9 (PMC8060298; doi:10.1038/s41598-021-88123-9)
Supplement: Supplementary file 1 — Supplementary Information 1. [file 41598_2021_88123_MOESM1_ESM.pdf]

**Supplementary information**

**Title:** Overexpression of the transcribed ultraconserved region Uc.138 accelerates colon cancer progression

**Authors:** Yuki Kuwano*, Kensei Nishida, and Kazuhito Rokutan

Department of Pathophysiology, Institute of Biomedical Sciences, Tokushima University Graduate School, Tokushima 770-8503, Japan

# Supplementary Table S1. Primer sets used for qPCR and cloning

| Primers | | |
|---|---|---|
| Targets | | Primer Sequences (5' - 3') |
| (for qPCR) | | |
| *TRA2B* | Forward (S1) | AGGAAAATGCGGAAGTCGTC |
| | Forword (S2) | CGGCGAGCGGGAATCCCG |
| | Reverse (AS1) | GACATGGGAGAATGGCTGTGGC |
| | Reverse (AS2) | CTGGAAGCAGAACGGGATTCTTA |
| *GAPDH* | Forward | AGCCACATCGCTCAGACAC |
| | Reverse | GCCCAATACGACCAAATCC |
| *CCNA1* | Forward | ACCCCAAGAGTGGAGTTGTG |
| | Reverse | GGAAGGCATTTTCTGATCCA |
| *CCNB* | Forward | CGGGAAGTCACTGGAAACA |
| | Reverse | AAACATGGCAGTGACACCAA |
| *CDKN1A* | Forward | GCAGGGGACAGCAGAGGAAG |
| | Reverse | AGAAGATCAGCCGGCGTTTG |
| (for cloning) | | |
| *TRA2B4 full-length* | Forward | AAAACTCGAGGTGCGGGACGCGCTGCAGCTGGA |
| | Reverse | AAAAGATATCTTACATTATATATTAAAGAT |
| *exon 2* | Forward | AAAACTCGAGGTTAATGTTGAAGAAGGAAAATGC |
| | Reverse | AAAAGATATCTTAGCGTAGTGCTTTCTGATTC |
| | | *The amplified fragment was subcloned into pEB-multi-Bsd (Wako) using XhoI and ExoRV sites (these sequences are underlined) |

**Supplementary Figure S1. Subcellular distribution of *TRA2B4* mutants in HCT116 cell clones.**

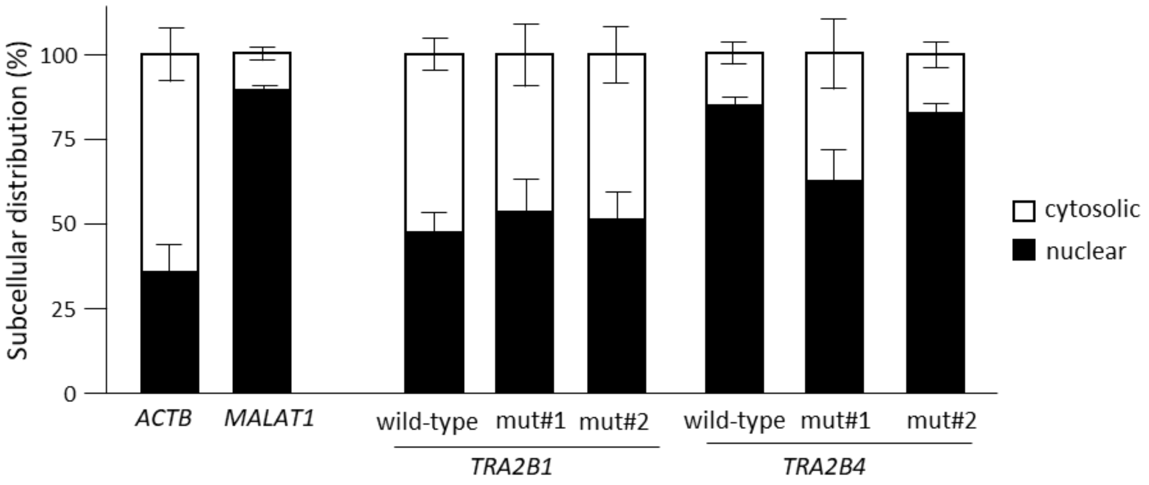


**Supplementary Figure S1.**
**Subcellular distribution of *TRA2B4* mutants in HCT116 cell clones.**

Bar graphs represent relative RNA quantity in cytosolic versus nuclear localization for wild-type or mutated *TRA2B4*. Levels of *ACTB* mRNA and *MALAT1* lncRNA in mock were used as controls to check purity of the cytosolic and nuclear fractions, respectively. RNAs from cytoplasmic or nuclear fraction were extracted, and the expression levels were measured by RT-qPCR as previously described[21].

**Supplementary Figure S2. The effects of *TRA2B4* mutations on resistance to apoptosis**

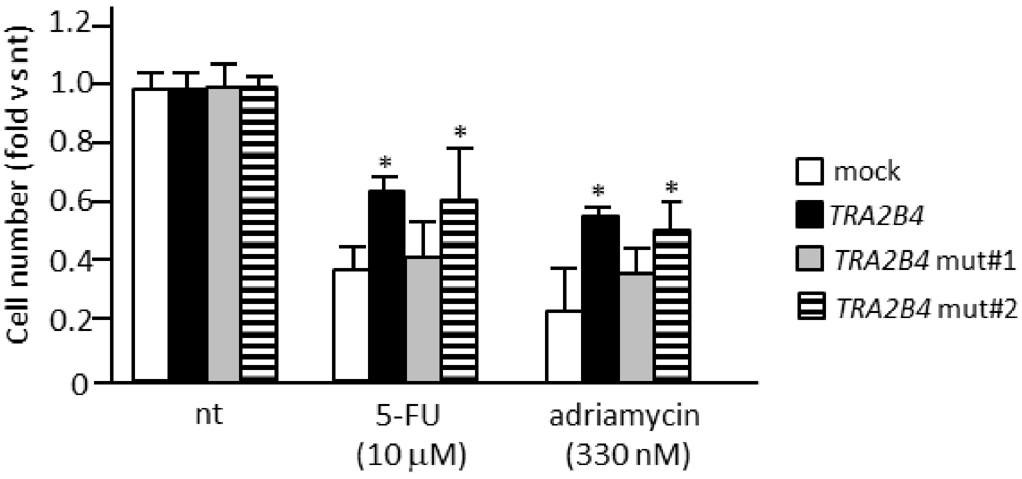


**Supplementary Figure S2. The effects of *TRA2B4* mutations on resistance to apoptosis**

HCT116 cells with stable overexpression of *TRA2B4* with or without mutations were exposed to 10 uM 5-fluorouracil (5-FU) or 330 nM adriamycin for 48 h. Subsequently, growing cells were harvested and counted. Cell viability was calculated as fold differences of non-treated cells. nt: non-treatment. Values are mean ± SD from four independent experiments. *Significantly different by the unpaired Student's t-test compared with mock-treated cells ($p < 0.05$).

**Supplementary Figure S3.** **Original blots from Figure 3E.**

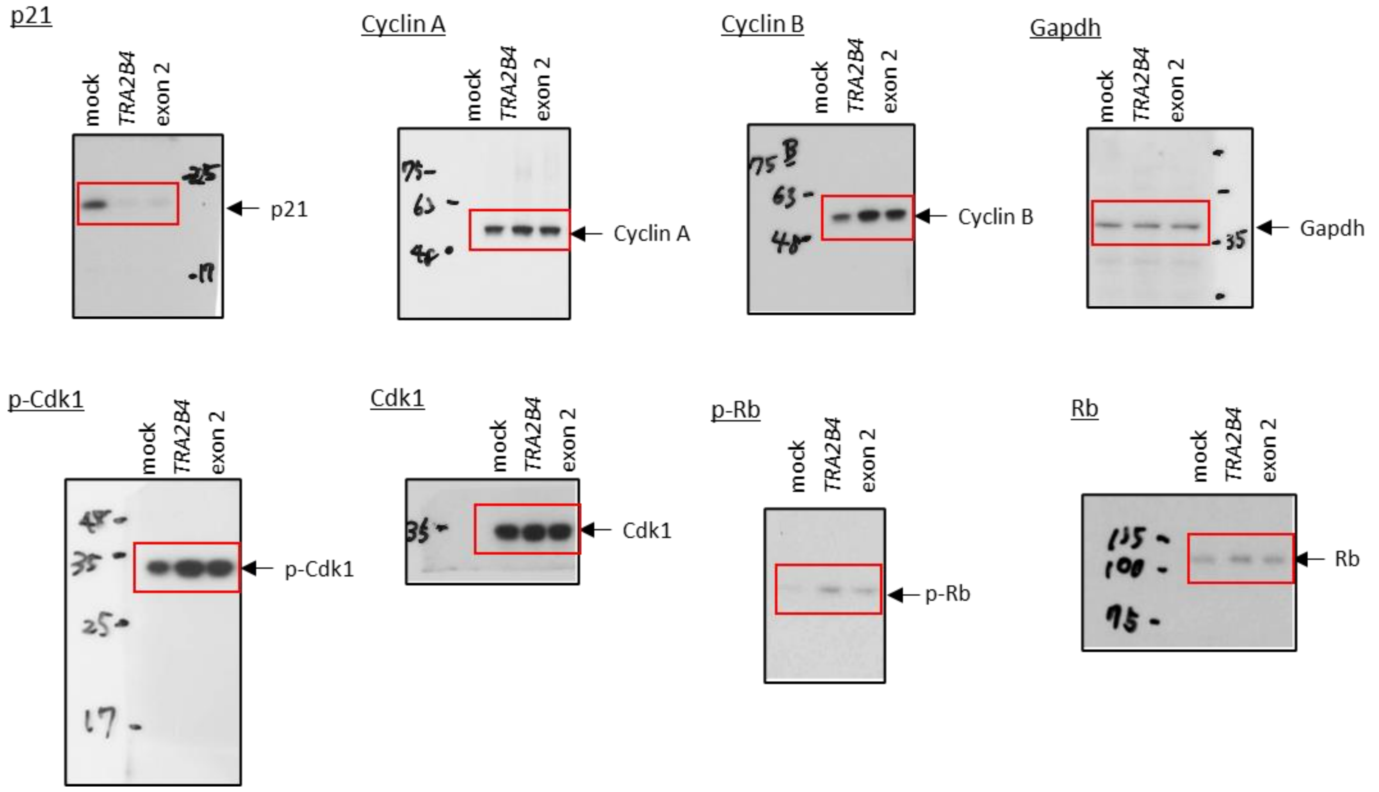


**Supplementary Figure S3.**
Amounts of cell cycle related proteins and phosphorylation of Cdk1 were determined using western blotting with Gapdh as a loading control as shown in Figure 3E. The target bands used in the figure are indicated by red square.
